# Acknowledgement to Reviewers 2022

**DOI:** 10.3389/phrs.2023.1605947

**Published:** 2023-03-16

**Authors:** PHR Editorial Office

**Affiliations:** Swiss School of Public Health (SSPH+), Zürich, Switzerland

The Editors of *Public Health Reviews* would like to thank all Reviewers in 2022 for the time they have spent and the valuable contributions they have made to ensure the scientific quality of the journal.

Carla AbouZahr, Switzerland

Ana Johnson, Canada

Gabriela Albuquerque, Portugal

Vishaldeep Kaur Sekhon, United States

Saleh Alessy, Saudi Arabia

June Kloubec, United States

Diogo Almeida, Portugal

Mark Kroese, United Kingdom

Isabel Alvarado-Cruz, United States

Thao Minh Lam, Netherlands

Alain Amstutz, Switzerland

Elizabeth Lee, United States

Daniela Anker, Switzerland

Jonathon Leider, United States

Saeed Anwar, Canada

Mayara Lima, Brazil

Joao Araujo, Brazil

Cosima Lisi, Portugal

Mark Avery, Australia

Laura Magaña, United States

Suzanne Babich, United States

Carlos Matos, Portugal

Barbara Bürkin, Switzerland

Miroslaw Mazurek, Poland

Simon Capewell, United Kingdom

Sinenhlanhla Memela, South Africa

Luisa Conceição, Portugal

Elina Miteniece, Netherlands

Ana Rute Costa, Switzerland

Samantha Morais, Canada

Ana Cruz, Portugal

Jessica Neicun, Netherlands

Valeria Carolina Cuenca Cuenca, Spain

Vasileios Nittas, United States

Katherine Curry, United States

Ngoy Nsenga, Switzerland

Katarzyna Czabanowska, Netherlands

Vicka Oktaria, Indonesia

Nicole Geovana Dias, Brazil

Oyeladun Okunromade, Nigeria

Rita Dias, Portugal

Rasaq Oladapo, Nigeria

Abeer Elshater, Egypt

Laura Orlando, Canada

Marta Fadda, Switzerland

Martina Paric, Netherlands

Rebecca Forman, United Kingdom

David Patterson, Netherlands

Cassiano Franco, Brazil

Elizabeth Peacocke, Norway

Xing Gao, United States

Margarida Pereira, Switzerland

Mark Green, United Kingdom

Priyanka Priyanka, United States

Mohammad Mehedi Hasan, Bangladesh

Ana Catarina Queiroga, Portugal

Elena Isaevska, Switzerland

Rajesh Kumar Rai, India

Kunihiro Iwamoto, Japan

Jesús Rivera Navarro, Spain

Cláudia Raquel Jardim Santos, Switzerland

Pablo Rodríguez-Feria, Netherlands

Neusa Jessen, Mozambique

Ankur Saxena, India

Vivi Schlunssen, Denmark

Florence Secula, Switzerland

Kenbon Seyoum, Ethiopia

Dawa Sherpa, India

Avaneesh Singh, India

Prashant Kumar Singh, India

Asha Soletti, India

Maoyi Tian, China

Cecilia Tinonin, Thailand

Yu-Chuan Tsai, Taiwan

Kaspar Wyss, Switzerland

